# Classification and Hemodynamic Characteristics of Uterine Artery Blood Flow in Recurrent Spontaneous Abortion

**DOI:** 10.2174/0115734056367096250508073203

**Published:** 2025-05-13

**Authors:** Yunyun Cao, Guanjie Wang, Haifei Wang, Ping Chen, Xiaoping Gong

**Affiliations:** 1 The International Peace Maternity and Child Health Hospital, School of Medicine, Shanghai Jiao Tong University, Shanghai 200030, China; 2 Shanghai Key Laboratory of Embryo Original Diseases, Shanghai 200030, China; 3 Institute of Birth Defects and Rare Diseases, School of Medicine, Shanghai Jiao Tong University, Shanghai 200030, China

**Keywords:** Recurrent spontaneous abortion, Doppler ultrasonography, Uterine artery, Pulsatility index, Hemodynamic parameters, Pregnancy complication

## Abstract

**Introduction:**

Recurrent spontaneous abortion (RSA) demonstrates a complex pathogenesis. The uterine artery (UtA) Doppler ultrasound monitoring is clinically valuable for predicting RSA risk.

**Objective:**

This study aimed to assess the type of blood flow velocity waveform (FVW) and the hemodynamic characteristics of the UtA between the RSA and control groups.

**Methods:**

This retrospective study included 203 patients with RSA and 121 without RSA. All participants underwent transvaginal Doppler ultrasonography during the mid-luteal phase to assess the type of FVW and the hemodynamic parameters of the UtA.

**Results and discussion:**

The C type was the most prevalent in both the control and RSA groups, with incidences of 80.16% and 63.04%, respectively. The single type was more predominant in the control group than in the RSA group (83.47% *vs*. 73.89%). Notably, the compound type was more frequent in the RSA group than in the control group (26.11% *vs*. 15.26%). The compound type exhibited significantly higher circulatory resistance than the single type, with significant statistical differences observed in the mean pulsatility index (mPI) and mean resistance index (mRI) between the two types (*P* < 0.001). Further, mPI and mRI values of the UtA were higher in the RSA group than in the control group, with significant statistical differences between the two groups (*P* < 0.001). If abnormal UtA hemodynamic parameters and FVW are detected, early clinical intervention should be implemented to improve adverse pregnancy outcomes.

**Conclusion:**

UtA FVW varies, indicating differences in blood resistance. Prepregnancy monitoring of high-resistance FVW and hemodynamic parameters effectively assessed uterine perfusion status and may provide a foundation for early clinical intervention and potential personalized treatment strategies.

## 1 INTRODUCTION

Abortion, including the biochemical and recessive types, is a prevalent pregnancy complication. Approximately 80% of abortions occur before 12 weeks of pregnancy. Spontaneous abortion (SA) affects 15%–25% of pregnancies, with recurrent SA (RSA) occurring in 1%–5% of cases. RSA significantly affects couples, specifically their physical, psychological, social, and economic well-being. RSA demonstrates a complex pathogenesis and involves single or multiple factors. Its etiology includes genetic, anatomical, endocrine, immunological, infectious, thrombotic, male, environmental, mental, pharmacological, nutritional, and unknown factors [1-5].

The uterine artery (UtA) is crucial for uterine blood perfusion, which is essential for maintaining endometrial receptivity and successful embryo implantation and development [6]. The UtA blood flow resistance in RSA has been elevated, which is deleterious to local vascularization of the endometrium and may cause uterine blood perfusion insufficiency, thereby affecting maternal and fetal blood circulation. Furthermore, the UtA blood flow resistance was markedly increased in patients with uterine malformations, antiphospholipid antibody syndrome, and unexplained RSA in comparison to the control group. This indicates that UtA blood flow insufficiency may be a key factor in the pathogenesis of RSA [7]. Color Doppler ultrasound is predominantly used in clinical practice to assess endometrial receptivity and monitor uteroplacental circulation. Monitoring UtA circulatory resistance parameters with Doppler ultrasound can predict RSA risk.

Based on the above research results, this study mainly aimed to investigate the classification of blood flow velocity waveform (FVW) and hemodynamic changes of the UtA in cases of nongenetic RSA while conducting a comparative analysis with a control group.

## 2 MATERIAL AND METHODS

### 2.1 Study Design and Participants

This retrospective case–control study enrolled patients who visited the prepregnancy clinic and the abortion clinic of the International Peace Maternity and Child Health Hospital of Shanghai Jiao Tong University School of Medicine from December 2018 to February 2022. A complete set of abortion-related tests were conducted in a standardized manner, including chromosomal examination of the couple, semen examination of the man, endocrine hormones and immunity of the woman, *etc*. The medical ethics committee of our hospital approved this study. Patients were informed about the study procedures to participate in the study, and each patient signed written informed consent.

Inclusion criteria were 1) complete clinical data, including patient age, menstrual history, fertility history, height, and weight and 2) satisfactory acquisition of UtA Doppler parameters.

Exclusion criteria were [8-12] 1) couples with chromosomal or genetic problems, 2) semen problems in males, 3) uterine malformations, 4) uterine adenomyosis, 5) uterine adhesions, 6) unmeasurable UtA, 7) primary hypertension, 8) diabetes, 9) endocrine abnormalities, and 10) patients already receiving medication (including aspirin, heparin, *etc*.).

### 2.2 Method of Measuring the UtA and Ultrasonographic Assessment

The UtA Doppler parameters were measured with a GE Voluson E8 ultrasound machine (GE Healthcare, Milwaukee, WI, USA) equipped with a 5–9 MHz transvaginal color Doppler probe. A single operator (Dr. Yunyun Cao) performed all Doppler measurements to ensure consistency.

The same operator (Yunyun Cao) conducted transvaginal ultrasound for all cases in the midluteal phase to avoid intraobserver variations. The sagittal section of the uterus was obtained, and the cervical canal was determined. The probe was then moved laterally until the paracervical vascular plexus was visualized. Color Doppler imaging was employed to identify the UtA as it ascended to the corpus uteri. The main ascending branch was measured before the arcuate artery separated from the UtA. Simultaneously, the angle of the control beam should be kept as parallel as possible to the direction of the blood flow or the correction angle should be <30°. At least five cardiac cycles were displayed on each image to obtain the blood flow spectrum diagram, and relevant blood parameters of both sides of the UtA were recorded, including peak systolic velocity, time-averaged velocity (TAV), end-diastolic velocity, resistance index (RI), and pulsatility index (PI), calculated by the formula RI = S-D/S; PI = S-a/TAV; mean PI (mPI) = (left-PI + right-PI)/2; mean RI (mRI) =(left-RI + right-RI)/2 [13]. Meanwhile, the diastolic phase of the bilateral UtA waveform was recorded for classification.

### 2.3 The FVW Classification of the UtA

**Fig.(1):**
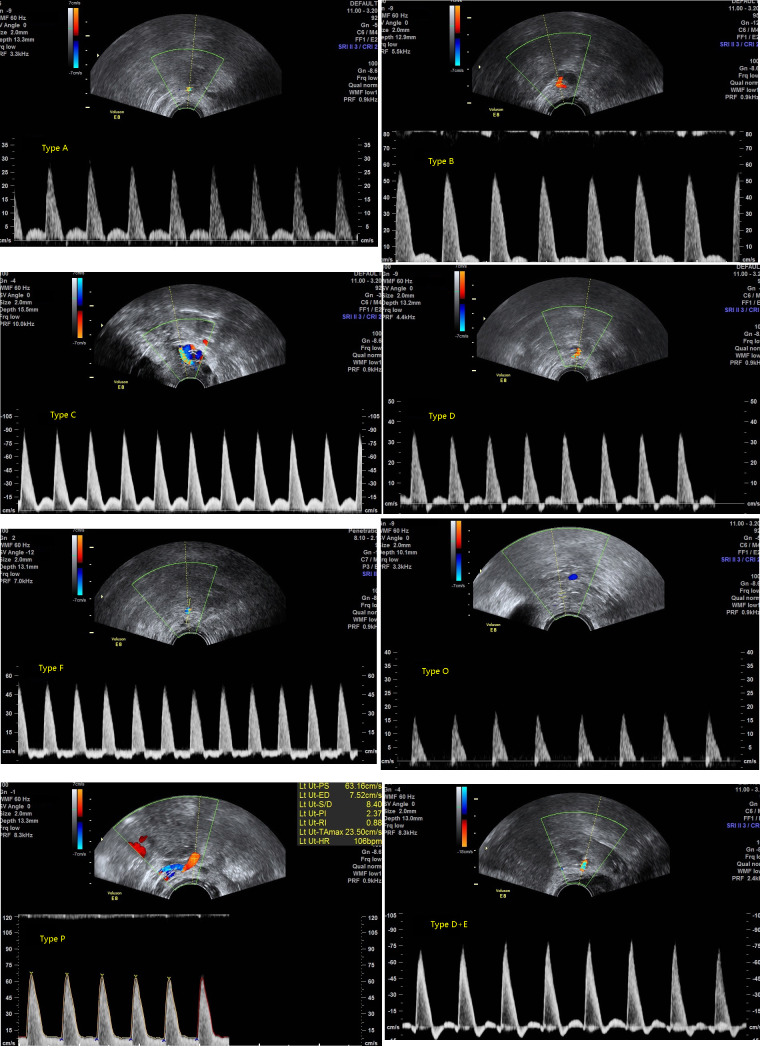
Uterine artery flow velocity waveform (FVW) classification. (From left to right and from top to bottom , Type A, Type B, Type C, Type D, Type F, Type O, Type P, Type D+E)

The FVW classification of the UtA was defined as follows: absence in early diastole was Type A, absence in end-diastole was Type B, early diastolic notch was Type C, early diastolic reverse was Type D, end-diastolic reverse was Type E, complete diastolic reverse was Type F, complete diastolic absent was Type O, and early diastolic no notch was Type P (Fig. **1**).

It is categorized based on the UtA FVW classification into a single type, wherein the classification of the bilateral UtA includes only one type, and a complex type, in which the classification of the bilateral UtA includes two or more types.

### 2.4 Statistical Analysis

MedCalc version 15.8 (MedCalc Software, Ostend, Belgium) was used for all statistical data analyses. Continuous variables that followed a normal distribution were expressed as mean (X̅) ± standard deviation (s). The Kolmogorov–Smirnov test or Shapiro–Wilk test was used to test normality, and Levene’s test was employed to assess homogeneity of variance. Comparisons between groups for normally distributed data were conducted using independent samples *t*-tests, as these are appropriate for detecting mean differences between two independent groups under these assumptions. Categorical data were expressed as N (%), and the Chi-square (χ^2^) test was used for comparisons between groups, considering its suitability for comparing proportions between groups. Data or tables with a large number of small cell frequencies were reorganized based on clinical expertise, where appropriate, by merging rows or columns in the contingency table to better reveal the association between the two variables. A two-sided *P*-value of <0.05 indicated statistical significance.

## 3 RESULTS

This study initially enrolled 343 cases and ultimately included 324 cases after excluding 19 cases. These exclusions included 2 cases with chromosomal abnormalities in either partner (1 Robertsonian translocation and 1 balanced translocation), 3 cases of thyroid dysfunction, 1 case each of unicornuate uterus and uterine septum, 1 case with an unmeasured UtA on one side, and 11 cases with incomplete medical records. The control group comprised 121 patients with no SA history (SA = 0), aged 23–45 years (mean 32.60 ± 3.87). The RSA group consisted of 203 patients with ≥2 SA a history (SA ≥ 2), aged 22–44 years (mean 32.82 ± 4.65). The frequency of SA ranged from 2 to 7. The delivery history of the control and RSA groups were 21.49% and 24.14%, respectively. The RSA group demonstrated slightly higher mean age and body mass index (BMI) than the control group (Table 1). However, no statistically significant difference in age, BMI, and reproductive history was found between the two groups (*P* > 0.05).

The C type was the most prevalent FVW in both the control and RSA groups (80.16% *vs*. 63.04%) Table 2. The control group (83.47%) demonstrated more prevalent single-type FVW than the RSA group (73.89%). Conversely, the RSA group (26.11%) exhibited more frequent compound-type FVW than the control group (15.26%). The RSA group demonstrated a higher proportion of diastolic blood flow velocity absent or reversal (*i.e*., Types A, B, D, E, F, and O) than the control group. Furthermore, approximately 98.63% of the compound types exhibited one type of A, B, D, E, F, and O, with a maximum of three types observed.

Table 3 presents that the compound-type FVW with a mean pulsatility index (mPI) of 3.20 ± 0.50 (95% confidence interval [CI]: 3.08–3.31) and a mean resistance index (mRI) of 0.93 ± 0.05 (95% CI: 0.93–0.96). In contrast, the single-type FVW exhibited an mPI of 2.08 ± 0.42 (95% CI: 2.03–2.14) and an mRI of 0.82 ± 0.06 (95% CI: 0.81–0.83). These results indicate a significantly higher circulatory resistance in the compound type than in the single type. Comparisons of mPI and mRI between the two types of UtA classification revealed statistically significant differences (*P* < 0.001). Further, comparisons of mPI and mRI between the single and compound types in both the control and RSA groups revealed significant statistical differences (*P* < 0.001).

The findings indicate that the FVW of the UtA exhibits diversity, and the different blood flow velocity waveforms during the diastolic period, to some extent, identify the varying resistance of the UtA. These differences indicate that the compound-type FVW is associated with higher blood flow resistance, which may impair uterine blood perfusion and contribute to RSA.

The mean mPI values in the control and RSA groups were 2.20 ± 0.64 (95% CI: 2.09–2.31) and 2.42 ± 0.38 (95% CI: 2.33–2.50), respectively Table 4. The mean mRI values in the two groups were 0.83 ± 0.07 (95% CI: 0.82–0.85) and 0.86 ± 0.07 (95% CI: 0.85–0.87), respectively. The RSA group demonstrated higher UtA mPI and mRI than the control group, with statistically significant differences observed between the two groups (*P* = 0.003 and *P* = 0.002, respectively). This statistical significance indicates a strong association between increased UtA resistance and RSA, emphasizing that higher mPI and mRI values could be used as predictive markers for RSA.

## 4 DISCUSSION

Current Chinese medical practice defines RSA as the loss of three or more pregnancies before the 28-week gestational mark, with a fetal weight of <1000 g. RSA is the clinical loss of two or more consecutive pregnancies. This definition is based on the observation that the recurrence rate after two consecutive miscarriages is similar to that after three consecutive miscarriages [10, 14, 15]. The risk of SA increases with the number of pregnancy losses. The miscarriage rate in the subsequent pregnancy can reach as high as 40%–80% after three consecutive SAs. Most early pregnancy losses are sporadic, with an estimated 50% incidence of chromosomal abnormalities in the embryo. However, the likelihood of chromosomal abnormalities decreases as the number of miscarriages increases. Further, earlier miscarriages are associated with a higher incidence of embryonic chromosomal abnormalities [14, 16].

In the normal menstrual cycle, the resistance of UtA to blood flow gradually decreases during the luteal phase, reaching its lowest level during the embryo implantation period. This is due to the high estrogen levels before ovulation, which dilates the UtA and reduces vascular resistance. Both estrogen and progesterone levels are increased in the midluteal phase, with progesterone relaxing the smooth muscles and further reducing UtA resistance. Increased UtA blood flow during this period develops a suitable environment for embryo implantation. Conversely, inadequate UtA blood flow during the luteal phase results in poor endometrial development, thereby hindering embryo implantation and causing SA. Therefore, monitoring UtA blood flow resistance during the midluteal phase in nonpregnant women has become a consensus [6]. Studies have revealed that high UtA resistance causes conditions such as preeclampsia and fetal growth restriction. Abnormal uterine perfusion reduces endometrium vascularization, thereby directly affecting uterine perfusion and maternal–fetal circulation, which may contribute to RSA [17-19]. Other studies indicate that in patients with RSA with increased UtA resistance, local endometrial vascularization is reduced, causing placental ischemia and thrombosis, thereby developing an unfavorable uterine microenvironment for embryo implantation and fetal development [20, 21]. This emphasizes the importance of a normal UtA blood supply for embryo growth.

Lazzarin *et al.* [7] classified patients with RSA based on etiology and revealed that the mPI was highest in uterine malformation cases, followed by antiphospholipid antibody syndrome and unexplained RSA, all of which were higher than the control group. Approximately 50% of patients with RSA have a thrombotic tendency, which is considered a significant cause of RSA. This is primarily due to increased coagulation and decreased fibrinolysis, resulting in thrombosis in the uterine spiral arteries or villous vessels, thereby causing poor placental perfusion and miscarriage. Studies on patients with unexplained RSA showed that the mPI in the antinuclear antibody-positive group was higher than in the control group, with PI values negatively correlated with serum progesterone levels [21, 22]. Wang *et al.* revealed that low-dose aspirin improves endometrial receptivity and significantly reduces endometrial and UtA circulatory resistance in patients with unexplained RSA [23]. These studies measured UtA circulatory resistance during the midluteal phase in nonpregnant patients, demonstrating significantly increased resistance in both unexplained RSA and RSA classified by etiology. Thus, increased UtA resistance during the midluteal phase may be both a cause and an intermediate factor or pathological manifestation of RSA.

Currently, the primary indicators of UtA hemodynamics include the FVW and resistance parameters (including S/D, PI, and RI). The S/D, PI, and RI reflect blood flow resistance, with PI representing resistance throughout the cardiac cycle and providing an overall assessment of the blood flow spectrum; however, the S/D and RI only reflect resistance at specific points. Therefore, this study used PI and RI to assess UtA circulatory resistance. Goswamy *et al.* [24] classified UtA FVW into Types A (absence of early and late diastolic flow), B (absence of end-diastolic flow), C (notch in early diastole with continuous flow until the end of the cardiac cycle), and O (complete absence of diastolic flow). Qingrong *et al.* [25] revealed that nonpregnant women with RSA predominantly demonstrated Type A and O waveforms, whereas the control group mainly exhibited Type C. Further, the RSA group showed significantly higher mPI than the control group. Mansour *et al.* [26] demonstrated that Type C was the most prevalent waveform in the control group (80%), followed by Type B (20%), whereas Type A or O was not detected. The RSA group demonstrated Type A as the most prevalent (60%), followed by Type O (20%), with Types B and C each accounting for 10% each. The results of our study were generally consistent with the findings of previous studies, with Type C being the most prevalent in the control group (80.16%), and mPI was significantly higher in the RSA group than in the control group. Further, this study did not adopt the aforementioned classification because the authors observed other FVW patterns, including reversed diastolic flow. Our study categorized FVWs into eight types based on clinical experience, with inconsistent bilateral UtA classifications, which were further classified into single or composite types. Ferreira *et al.* [27] reported a significantly higher mPI in the RSA group than in the control group, with a receiver operating characteristic curve analysis revealing that an mPI cutoff value of 3 had a sensitivity of 34.9% and specificity of 93.0% for predicting RSA recurrence.

Goswamy *et al.* [24] hypothesized that UtA perfusion decreases from Type C to Type O, with lower diastolic flow indicating higher resistance and poorer uterine perfusion. The FVW simplified the assessment of UtA perfusion. In this study, absent or reversed end-diastolic flow (Types B, E, F, and O) indicated high distal resistance and insufficient perfusion. Two Type O and one Type F cases in the RSA group demonstrated high-resistance indices (mPI > 3 and mRI ≥ 1). RI depends on the end-diastolic flow velocity, so absent or reversed early diastolic flow (Types A and D) may not necessarily cause high RI or PI. When performing UtA assessments in clinical practice, we not only document quantitative parameters but also classify the FVW waveform patterns. This waveform classification provides a valuable reference for clinical treatment assessment. Drug therapy that successfully converts Type B, O, E, or F waveforms back to Type C or P patterns can serve as one of the indicators for treatment efficacy.

Different FVW can qualitatively indicate the circulatory resistance of UtA as demonstrated by the above analysis, in addition to the quantitative indicators PI and RI, which directly reflect the circulatory resistance of UtA. The results of this study are roughly comparable to the findings of the previous research but with some differences. This may be because of different examination routes (transabdominal or transvaginal), sample sizes, selected populations, inclusion and exclusion criteria, and subtle differences in the UtA FVW classification. Further, variations in Doppler ultrasound techniques or sample heterogeneity may be potential confounding factors affecting the monitoring results. UtA Doppler ultrasound measurements are influenced by operator bias, which may affect measurement accuracy, introduce measurement bias, and increase random errors. To improve data reliability, the same experienced sonographer performed all Doppler assessments in our study using a standardized protocol. This approach minimized interoperator variability, improved measurement accuracy, and reduced both measurement bias and random errors between individuals.

In recent years, the understanding of RSA-related blood resistance factors has improved, and monitoring UtA resistance during the luteal phase has gained attention as a screening tool. If an abnormality is found, early intervention and treatment should be given to reduce it to the normal range before pregnancy or early in pregnancy can significantly improve pregnancy outcomes. Concurrently, the UtA circulating RI can be used as one of the monitoring indicators for RSA treatment to monitor uterine blood perfusion.

This study has some limitations. The sample size was not large enough to stratify patients with RSA based on different etiology. In future work, we will further expand the sample size and integrate machine learning techniques with longitudinal data to develop predictive models for RSA [28, 29]. Using advanced computational methods, we aim to automate the UtA FVW classification patterns and enable early high-risk patient identification [30]. Further, longitudinal studies could elucidate the complex interactions between genetic, immunological, and environmental factors in RSA, thereby providing a better understanding of its etiology and facilitating the development of targeted interventions to reduce the occurrence of miscarriage.

## CONCLUSION

In summary, this study primarily analyzed the distribution of UtA FVW in the control group and patients with RSA. UtA FVW exhibits diversity, and to some extent, the differences in diastolic blood flow velocity waveforms identify the variations in the UtA resistance. The proportion of compound types was higher in the RSA group than in the control group. Compound FVW may indicate higher circulatory resistance to a certain extent. The single Type C was the most prevalent FVW in both groups; however, the proportion of compound types was significantly higher in the RSA group. Further, the mPI and mRI values were higher in the RSA group than in the control group. Therefore, FVW that exhibits the absence or reversal of diastolic blood flow (*i.e*., Types A, B, D, E, F, or O in this study) or has higher mPI and mRI values may indicate increased UtA circulatory resistance and decreased blood perfusion, emphasizing a higher risk of recurrent miscarriage. Thus, the authors recommend that UtA circulatory resistance monitoring before pregnancy should not only be performed in patients with RSA but also in those who have experienced one SA without embryonic chromosomal testing. This enables timely prevention or treatment, thereby reducing the risk of RSA and avoiding further psychological and physical trauma.

## AUTHORS’ CONTRIBUTIONS

P.C., X.G.: Study conception and design; H.W.: Data collection; G.W.: Analysis and interpretation of results; Y.C.: Draft manuscript.

## Figures and Tables

**Table 1:** Comparison of basic clinical data between the control group and RSA group.

-	**Control Group (N=121)**	**RSA Group (N=203)**	**P-value**
Age(y)	32.60±3.87	32.82±4.65	0.861
BMI(kg/m^2^)	21.63±2.62	22.32±3.19	0.105
Parity (N/%)	-	-	0.585
No	95(78.81)	154(75.76)	-
Yes	26(21.49)	49(24.14)	-
Spontaneous abortion (N/%)	-	-	-
0	121(100)	0(0)	-
1	0(0)	0(0)	-
2	0(0)	142(69.95)	-
3	0(0)	49(24.14)	-
4	0(0)	6(2.96)	-
5	0(0)	3(1.48)	-
6	0(0)	1(0.49)	-
7	0(0)	2(0.98)	-

**Table 2:** Comparison of UtA doppler waveforms on both sides of the control group and RSA group.

**Type **	**Control Group (N=121 )**	**RSA Group (N=203 )**	**P-value**
Single type (N/%)	101(83.47)	150(73.89)	0.045^a^
A	2(1.65)	2(0.99)	-
B	1(0.83)	1(0.49)	-
C	97(80.16)	145(63.04)	-
D	0(0)	1(0.49)	-
P	1(0.83)	1(0.49)	-
Compound type (N/%)	20(15.26)	53(26.11)	-
A+B	2(1.65)	8(3.94)	-
A+B+C	5(4.13)	8(3.94)	-
A+B+D	2(1.65)	4(1.97)	-
A+C	4(3.31)	11(5.4)	-
A+C+D	0(0)	1(0.49)	-
A+C+O	0(0)	1(0.49)	-
A+D	2(1.65)	4(1.97)	-
B+C	0(0)	4(1.97)	-
B+C+D	0(0)	5(2.5)	-
B+D	2(1.65)	1(0.49)	-
B+D+E	1(0.83)	0(0)	-
C+D	1(0.83)	1(0.49)	-
C+D+E	0(0)	1(0.49)	-
C+E	1(0.83)	0(0)	-
C+P	0(0)	1(0.49)	-
D+E	0(0)	1(0.49)	-
D+F	0(0)	1(0.49)	-
O+C	0(0)	1(0.49)	-

**Note**:RSA, recurrent spontaneous abortion. UtA, uterine artery. ^a^ represents the comparison between the single type and the compound type. By combining the data with professional knowledge, type B and type D in the single type are classified into the compound.

**Table 3:** Comparison of mPI and mRI in different types of bilateral UtA Doppler waveforms.

	**Control Group (N=121)**				**RSA Group (N=203)**				**Single Type (N=248)**		**Compound Type** **(N=76)**		**P-value**
	**Single type(N=100)**		**Compound type** **(N=21)**		**Single type(N=148)**		**Compound type** **(N=55)**						-
	x-±s	95%CI	x-±s	95%CI	x-±s	95%CI	x-±s	95%CI	x-±s	95%CI	x-±s	95%CI	-
mPI	2.00±0.43	1.91-2.08	3.30±0.63	3.01-3.59	2.14±0.41	2.07-2.21	3.15±0.44	3.04-3.27	2.08±0.42	2.03-2.14	3.20±0.50	3.08-3.31	<0.001
mRI	0.81±0.06	0.80-0.82	0.95±0.06	0.93-0.98	0.83±0.05	0.82-0.84	0.94±0.05	0.93-0.95	0.82±0.06	0.81-0.83	0.93±0.05	0.93-0.96	<0.001

**Note**: RSA, recurrent spontaneous abortion; mPI, mean pulsatility index; mRI, mean resistance index. By combining the data with professional knowledge, type B and type D in the single type are classified into the compound type.

**Table 4:** Comparison of UtA Doppler parameters mPI and mRI between control group and RSA group.

	**Control Group (N=121)**		**RSA Group (N=203)**		**P-value**
	x-±s	95%CI	x-±s	95%CI	-
mPI	2.20±0.64	2.09-2.31	2.42±0.38	2.33-2.50	0.003
mRI	0.83±0.07	0.82-0.85	0.86±0.07	0.85-0.87	0.002

**Note**: RSA, recurrent spontaneous abortion; mPI, mean pulsatility index; mRI, mean resistance index.

## Data Availability

All data generated or analyzed during this study are included in this published article.

## LIST OF ABBREVIATIONS

FVW: Flow velocity waveform
UtA: Uterine artery
RSA: Recurrent spontaneous abortion
BMI: Body mass index
PI: Mean pulsatility index
mRI: Mean resistance index
TAV: Time-averaged velocity
RI: Resistance index

## STANDARDS OF REPORTING

STROBE guidelines were followed.
